# Mediating role of resilience in the relationships of physical activity and mindful self-awareness with peace of mind among college students

**DOI:** 10.1038/s41598-023-37416-2

**Published:** 2023-06-27

**Authors:** Yu-Chi Liao, Tzu-Yun Huang, Szu-Hung Lin, Chia-Huei Wu, Kun-Tang Chang, Shulan Hsieh, Sheng-Hsiang Lin, Joshua Oon Soo Goh, Cheng-Ta Yang

**Affiliations:** 1 Department of Psychology, College of Medical and Health Sciences, Asia University, No. 500, Lioufeng Rd., Wufeng Dist., Taichung, 41354 Taiwan; 2 Center for Prevention and Treatment of Internet Addiction, Asia University, Taichung, Taiwan; 3 Clinical Psychology Center, Asia University Hospital, Taichung, Taiwan; 4 Department of Psychology, National Cheng Kung University, Tainan, Taiwan; 5 Department of Psychology, School of Science, Soochow University, Taipei, Taiwan; 6 Department of Human Resource Management and Employment Relations, King’s Business School, King’s College London, London, UK; 7 Department of Medical Research, China Medical University Hospital, China Medical University, Taichung, Taiwan; 8 International Doctoral Program in Principles and Implications of Mind Sciences, National Cheng Kung University, Tainan, Taiwan; 9 Institute of Allied Health Sciences, National Cheng Kung University, Tainan, Taiwan; 10 Institute of Clinical Medicine, College of Medicine, National Cheng Kung University, Tainan, Taiwan; 11 Department of Public Health, College of Medicine, National Cheng Kung University, Tainan, Taiwan; 12 Biostatistics Consulting Center, National Cheng Kung University Hospital, College of Medicine, National Cheng Kung University, Tainan, Taiwan; 13 Graduate Institute of Brain and Mind Sciences, College of Medicine, National Taiwan University, Taipei, Taiwan; 14 Graduate Institute of Health and Biotechnology Law, Taipei Medical University, No. 250, Wuxing St., Xinyi Dist., Taipei, 110 Taiwan

**Keywords:** Human behaviour, Psychology, Quality of life

## Abstract

Peace of mind (PoM) is an index of mental health in Asian culture and emphasizes low arousal, happiness, harmony, and an internal state of peacefulness. While previous studies have found that mindful self-awareness can contribute to PoM, regular physical activity (PA) is also an important factor contributing to one’s PoM due to its function in promoting one's resilience. The study aims to investigate a hypothetical model that assumes PA is associated with resilience while controlling for mindful self-awareness, contributing to PoM. The PoM scale, Connor-Davidson Resilience Scale, Chinese translation of Mindful Attention Awareness Scale, and PA self-report questionnaire were used. A path analysis was applied to test the association between these variables and the mediating role of resilience. A total of 436 students from a university in Taiwan were recruited; the mean age was 20.87, with 46.3% female and 73.6% engaging in over 150 min/week of moderate PA. Gender and age negatively correlated with PA. After controlling for age and gender, there was no direct effect of physical activity on PoM; both mindful self-awareness and PA predict resilience, which in turn predicts PoM, suggesting that both cognitive (i.e., mindful self-awareness) and PA are important to cultivate resilience and thus PoM.

## Introduction

Peace of mind (PoM) is an internal state of peacefulness and harmony. It represents an affective state valued in Chinese culture and the Chinese philosophies of Confucianism, Taoism, and Buddhism, emphasizing low-arousal positive affect and a harmonious state of happiness^1^. PoM has become an essential issue in emotional health^2^, especially in Asian culture^3^. Research has shown that Chinese individuals value low-arousal positive affect (i.e., calm and peace) more than European Americans^4^. The inner peace, calm, and low-arousal positive state, so-called PoM states, play critical roles in dealing with the climate and global changes^1,3^. There have been studies on low-arousal positive affect (i.e., inner peace and serenity) and its relationship with psychological well-being. For example, McManus et al.^5^ reported that in an adult sample (ages 20–79), low-arousal positive affect is associated with lower levels of depression, anxiety, and stress, and increased life satisfaction. Kreitzer et al.^6^ reported that serenity has a predictive effect on quality of life. In addition, many studies have demonstrated the positive effect of PoM on one's psychological well-being^7^. Yet, it remains unclear about the factors that may affect one's PoM.

To investigate antecedents of PoM, Lee et al. suggested that individuals are required to control their desires to comply with the critical virtue of benevolence and maintain equilibrium, a peaceful mean state without overly positive or negative emotions^1^. Their findings revealed that the construct of PoM effectively captures affective experiences that are highly valued within Chinese cultural contexts and provides an index of affective well-being. However, assessing PoM might be susceptible to the influence of an individual's attitudes toward the experience of PoM. People who prioritize the internal state of peace of mind over hedonic happiness may be more attentive to this state, and consequently, may report a higher frequency of peace of mind than those who value it less. In line with this view, the study has examined the role of mindful self-awareness in facilitating PoM. The concept of "mindfulness" originated from Buddhist meditation, emphasizing awareness of the present moment with a nonjudgmental attitude^8^. Mindfulness practice is a type of meditation involving breathing techniques, imagery, and other practices that cultivates present moment awareness nonjudgmentally^9^. That aim to foster intentional, nonjudgmental attention, are linked to self-regulation mechanisms and improvements in mental health, contributing to enhanced subjective well-being^10^. One's mindful self-awareness has been found to be positively associated with psychological well-being^11^ and negatively associated with undesired psychological outcomes, including emotional distress^12^, anxiety^13^, and general distress^14^. Regarding PoM specifically, studies have reported a positive association between practicing mindfulness (to improve mindful self-awareness) and PoM^15,16^. Xu et al.^15^ studied 212 university students and discovered a mediating effect of self-acceptance between mindfulness and PoM. Ge et al.^16^ analyzed a sample of 366 individuals aged 18–55 and found a positive correlation between mindfulness, resilience, and inner peace. Their findings suggest that incorporating mindfulness practice and reducing past-negative time perspectives could help develop new psychological interventions to address mental health issues such as negative bias, rumination, depression, anxiety, and PTSD. Liu et al.^17^ did a randomized controlled trial with 57 participants (78% were university students), suggesting the effectiveness of mindfulness training in improving inner peace among participants without known mental disorders. These findings suggested that mindfulness practice can facilitate one's inner peace by increasing acceptance, reperceiving, and insight into impermanence^15–17^.

Resilience may play a role as an antecedent of PoM. Resilience is the manifestation of positive outcomes in the face of significant threats or impediments to adaptation or developmental progression. It is more aptly described as a dynamic process that promotes positive adaptation within the context of pronounced adversity. The process hinges on two aspects: an individual's exposure to significant adversity and their successful adaptation despite these profound developmental challenges. Thus, resilience not only underscores an individual’s capacity to recover and sustain functionality amidst adversity but also embodies the process of successfully navigating through significant impediments to one's adaptive growth or development. This concept is intrinsically linked to commonplace protective processes, such as an individual's ability to regulate emotions and effective external environmental factors^18^. In the view of Connor and Davidson, resilience "embodies the personal qualities that enable one to thrive in the face of adversity (p.76)". It consists of dimensions such as personal competence, trust, positive acceptance, control, and spiritual influence. Resilience can contribute to PoM by increasing one's tolerance of negative affect and positive acceptance of change^19,20^. Kelley and Pransky^21^ proposed a perspective on trauma and human resilience based on three principles: mind, thought, and consciousness. The mind represents the formless energy flowing through all individuals; thought refers to the mind-powered ability to create psychological experiences from within; and consciousness denotes the mind-powered capacity to experience life and be aware of the psychological experiences created through thought. According to this understanding, individuals create their moment-to-moment psychological experiences from within, and these experiences are animated and made to appear "real" by consciousness. This perspective posits that individuals can access resilience when their minds clear, facilitating a shift in consciousness that fosters healing. By recognizing their innate resilience and cultivating a mindful thought process, people can incorporate it into their lifestyle, viewing negative thoughts as cues to let the mind clear rather than continuing to ruminate.

As such, resilience has been regarded as an endophenotypic factor, and it plays a critical role between the body (e.g., genotype, body condition)^22^, mind (e.g., cognitive flexibility), and mental health and well-being^16,23^. Empirically, resilience has been found to be beneficial to one’s mental health ^24–26^, such as better life satisfaction, subjective well-being ^27^, inner peace ^16^, and mental health ^28,29^. Studies of the effect of resilience on PoM are still rare. However, Xu et al. has found that self-acceptance, a factor of resilience^15^, can contribute to PoM.

Physical activity is crucial not only for physical health, but it may also have a significant influence on psychological outcomes, which is the focus of our research. Studies found regular physical activity (PA) can improve one's resilience by inducing better neuroplasticity, increases cognitive reserve, and improve self-immunity and reduce respiratory symptoms^30,31^. These physiological changes can promote better management of stress^32^ and mental condition^33^. For example, a study found that increased PA may facilitate self-regulation and resilience by improving one's ability to exert top-down control^34^. The greater frequency of moderate-to-vigorous intensity PA related with more positive psychosocial outcomes of grit and resilience^24,25,35,36^. Furthermore, studies examining college students’ PA^36^ and adults with inflammatory bowel disease^37^ have both suggested that increasing volumes of moderate or moderate-to-vigorous PA are associated with greater resilience, improved emotional management, and lower rates of psychological distress^36,37^. Additionally, Ho et al.^38^ have proposed a mediation model showing Chinese adolescent’s PA affected mental well-being through the mediation of resilience. However, they only used a single-item question to evaluate the students' average weekly physical activity level over the past year. Previous research has consistently demonstrated that physical activity plays a significant role in enhancing psychological outcomes and PoM, particularly by bolstering resilience.

Based on the above reasoning, the goal of this study is to examine whether PA can contribute to PoM through its influence on resilience. We hypothesize that PA indirectly contributes to PoM by bolstering resilience. To test this indirect effect, we propose a set of hypotheses. As previous studies have reported the positive effect of mindful self-awareness on resilience^39^ and PoM^15,17^, we will control for its effect when we examine our hypotheses. Specifically, we included mindful self-awareness as a research variable that will predict both resilience and PoM.

### Hypothesis 1

Physical activity is positively associated with resilience.

### Hypothesis 2

Resilience is positively associated with peace of mind.

### Hypothesis 3

Resilience mediates the association between physical activities and peace of mind.

## Methods

### Participants and procedure

A total of 476 participants (210 female, 267 male), aged 18–30 years, were recruited from the National Cheng Kung University community. The recruiting span from October 2017 to June 2018, which consist of two whole semesters. The participants were recruited through in-class invitations (approximately 589 students across two semesters of introductions in psychology), on-campus, and online advertising. To avoid confounding during recruitment, trained assistants maintain standard procedures while the principal investigator monitors and controls for potential confounding variables. The participants included first-year to sixth-year students from different colleges, including the medical college, college of humanities and social sciences, college of science, and college of engineering. Participants were administered a set of paper–pencil questionnaires by trained assistants and were reimbursed NTD160 per hour. The assessment took place in Room 536, Fifth Floor, North Building, Academy of Social Sciences, National Cheng Kung University. To reduce the dropout rate, all recruited participants were fully informed about the study details and potential risks, and written consent was obtained prior to the start of the experiment. The questionnaire completion process required approximately 40–60 min of the participants’ time. The assessment could be conducted either as a group or an individual test. Moreover, the testing room was equipped with sufficient desks and partitions to prevent any disturbances that could affect the participants' concentration. The questionnaires used in this study are paper-based, with Likert-type scales measuring different psychological characteristics. Ethical approval for this study was obtained from the Ethics Committee of the Department of Psychology at National Cheng Kung University (ethics code 108–072). The study was conducted with the approved guidelines and regulations.

We removed 40 participants who have more than 10% missing responses in the survey to avoid having data from careless respondents. A total of 436 participants (approximately 74% of two-semester courses) with a mean age of 20.87 (*SD* = 1.98) years, and the mean education in years was 14.98 (*SD* = 1.99). Among them, 202 were biologically female (46.33%), and 321 (73.6%) reported more than 150 min of moderate physical activity per week calculated by physical activity self-report. The means of the scores of scales were shown in Table 1.

**Table 1:** Descriptive statistics and correlations between all variables.

Scales	M	SD	Skewness	Kurtosis	Gender	Age	Physical activity	Mindfulness	Resilience	Peace of mind
1. Gender (female = 1)	202^a^	46.3^b^	–	–	–					
2. Age	20.87	1.98	1.531	4.069	− .015	–				
3. Physical activity (0 = under 150 min/week; 1 = over 150 min/week)	321^a^	73.6^b^	–	–	− .143**	− .132**	–			
4. Mindfulness	58.59	11.42	− .189	.664	− .003	.067	− .016	–		
5. Resilience	63.34	14.50	− .116	− .058	− .051	.063	.164**	.323***	–	
Resilience: Personal Competence	2.55	0.70	− .180	− .216	− .073	.017	.137**	.289**	.934**	.576**
Resilience: Trust	2.53	0.58	− .064	− .055	− .114*	.061	.169**	.210**	.872**	.523**
Resilience: Positive acceptance	2.66	0.65	− .254	− .018	− .007	.101*	.154**	.356**	.868**	.626**
Resilience: Control	2.33	0.83	− .061	− .559	.015	.055	.101*	.318**	.823**	.585**
5.5 Resilience: Spiritual Influence	2.48	0.78	− .190	.048	.077	.064	.107*	.173**	.554**	.335**
Peace of mind	3.38	.77	− .126	− .298	− .047	.055	.121*	.331***	.645***	–

N = 436, ^a^N; ^b^percentage; *p < .05; **p < .01; ***p < .001.

### Measures

To assess the participants’ state of mind, the Connor-Davidson Resilience Scale (CD-RISC), Chinese Translation of the Mindful Attention Awareness Scale (CMAAS) and Peace of Mind (PoM) Scale were used.

### Physical activity self-report

For physical activity measure, we designed a simplified version of the questionnaire based on the American College of Sports Medicine (ACSM) physical activity assessment criteria^40,41^. The questionnaire items include exercise frequency, exercise duration, and exercise intensity. The volume of physical activity was reported as minutes per week based on the number of days reported and the time spent engaged in the various exercise intensities. According to the criteria for physical activity of adults were recommended by World Health Organization (WHO), ACSM, and the American Medical Association (AMA) to be 150 min of moderate physical activity per week.

In this study, the self-report physical activity was assessed across four items: exercise habits, frequency, duration, and intensity. The adopted item and coding scheme were as follows: (item 1) exercise habits—absent (0), present (1); (item 2) exercise frequency—none (0), 1–2 times/week (1), 3–4 times/week (2), more than 5 times/week (3); (item 3) each exercise duration—less than 30 min/session (0), 31–60 min/session (1), 61–90 min/session (2), over 91 min/session (3); (item 4) intensity of exercise—low (0), moderate (1), vigorous (2). Consistent with ACSM and AMA guidelines, participants' weekly PA was determined by multiplying the weekly exercise frequency and duration. If the product of exercise frequency and duration was 4, 6, or 9, and the exercise intensity was 1 or 2, it was deemed to meet the ACSM and AMA recommendations for PA, thereby coded as 1 (or 0 if the criteria were not met). This coding identified participants who achieved over 150 min of moderate PA per week.

The overall reliability of the assessment, measured using the Cronbach's α coefficient, was 0.712. A single-factor analysis as validity examination yielded a Kaiser–Meyer–Olkin (KMO) measure of 0.667 and a significant Bartlett's test of sphericity (*p* < 0.001), explaining 56.339% of the total variance.

### Connor-Davidson resilience scale (CD-RISC)

We used the Connor-Davidson Resilience Scale (CD-RISC) to measure participants’ psychological resilience and their ability to thrive in the face of adversity Conner and Davidson^19^. The questionnaire consists of 25 items, rated on a scale from 0 to 4, ranging from “not true at all” to “true nearly all the time.” Sample items included “I am able to adapt when changes occur” and “Even when hopeless, I do not give up” The questionnaire encompasses five dimensions: personal competence, trust, positive acceptance, control, and spiritual influence. Greater resilience is indicated by higher scores. The Chinese variant of the CD-RISC was established by Wang et al. Their study involved 116 participants, aged 20 to 60 years, who were diagnosed with chronic mental illnesses and were receiving treatment at the Tsaotun Psychiatric Center. The internal consistency of this version was verified with a Cronbach's α score of 0.953. To evaluate test–retest reliability, the scale was re-administered to 47 out of the original 116 patients after an interval of two weeks. The resulting Pearson correlation coefficient was 0.798^42^. The alpha reliability (α = 0.929) was adequate in the current sample.

### Chinese translation of the mindful attention awareness scale (CMAAS)

The original Mindful Attention Awareness Scale (MAAS) was developed by Brown and Ryan^43^, and the Chinese version was created by Chang et al.^9^ through three studies. Exploratory (Study 1, n = 157) and confirmatory (Study 2, n = 204) factor analyses confirmed a one-factor solution. The scale demonstrated good internal consistency and test–retest reliability (Study 3, n = 116). CMAAS consists of 15 items scored on a 6-point Likert scale, ranging from "almost always" to" almost never.” Sample items included "I tend not to notice feelings of physical tension or discomfort until they really grab my attention" and "It seems I am ''running on automatic" without much awareness of what I'm doing." The scale measures mindful self-awareness, also known as mindfulness, and the ability to pay and maintain attention to present-moment experiences^9,43^. A higher score on the CMAAS represents higher mindful self-awareness. The Cronbach's alpha of the CMAAS in the Chang et al.^9^ 3 studies were 0.86-0.90. The alpha reliability (α = 0.834) was adequate in the current sample.

### Peace of mind scale (PoMS)

PoMS is a 7-item scale developed by Lee et al.^1^ that measures participants’ internal peace and well-being on a 5-point rating scale, ranging from" not at all" to" all of the time. " Sample items included " My mind is free and at ease" and " The way I live brings me feelings of peace and comfort. " ﻿ The alpha reliability coefficient of the PoM was 0.91^1^. The alpha reliability (α = 0.938) was adequate in the current sample.

### Data analysis

Before the data analysis, we checked and coded every single participant's questionnaire manually by two authors. After a comprehensive review of data, we excluded 40 participants who exhibited more than 10% missing responses in the survey. Following this removal, our final dataset did not contain any outliers, ensuring the robustness and validity of our findings.

Descriptive statistics were done to check each variable's mean, standard deviation, skewness, and kurtosis. Pearson's product–moment correlation coefficients were employed to examine bivariate associations, which were used to investigate the relationship between demographic variables (such as age and gender), physical activity, mindful self-awareness, resilience, and PoM. Then we conducted a multiple regression model to defined effect of each predictor. Furthermore, we conducted a path analysis to examine the mediating role of resilience in the relationships between physical activity, mindful self-awareness, and PoM. The adequacy of the model was analyzed with path analysis using AMOS 22 software. The path models were estimated using maximum likelihood and Huber's robust standard error estimator to account for the multilevel data structure^44^. Bootstrapping for mediation effects was used for factors predicted PoM^44^. The goodness-of-fit of the model was evaluated based on multiple indices, the cut points were as below. Root-Mean-Square Error of Approximation (RMSEA) and the value of Standardized Root-Mean-Square Residual (SRMR) below 0.05 indicated a good fit and values as high as 0.08 represented reasonable errors of approximation in the population^45^. The Comparative Fit Index (CFI) and Non-Normed Fit Index (NNFI) with values greater than 0.90 are considered a good fit^46^.

## Results

Table 1 presents descriptive statistics for the sample's gender, age, physical activity, mindful self-awareness, resilience, subscales of resilience (including personal competence, trust, positive acceptance, control, and spiritual influence) and PoM. The number of females was 202 (46.3%). The mean age was 20.87 (*SD* = 1.98). The number of participants who had more physical activity over 150 min/week was 321 (73.6%). The mean and standard deviation of mindful self-awareness, resilience, PoM and subscales of resilience (personal competence, trust, positive acceptance, control, and spiritual influence) were 58.5 (*SD* = 11.42), 63.34 (*SD* = 14.50), 3.38 (*SD* = 0.77), and subscales of resilience (2.55 (*SD* = 0.70), 2.53 (*SD* = 0.58), 2.66 (*SD* = 0.65), 2.33 (*SD* = 0.83), 2.48 (*SD* = 0.78)) respectively.

Additionally, the Pearson's product–moment correlation coefficients among the variables were calculated. Gender and age were negatively correlated with PA (*r* = -0.143, *p* < 0.01; *r* = -0.132, *p* < 0.01, respectively). Gender was negatively correlated with trust subscale of resilience (*r* = − 0.114, *p* < 0.05). Age was correlated with Positive acceptance subscale of resilience (*r* = -0.101, *p* < 0.05). Females and older participants were less likely to have more physical activity. Females showed lower score on trust subscale of resilience. Older participants showed more score on positive acceptance subscales. PA had a significant association with resilience (*r* = 0.164, *p* < 0.01) and its five subscales (*r* = 0.101–0.169) as well as PoM (*r* = 0.121, *p* < 0.05). Mindful self-awareness was found to have a significant association with resilience (*r* = 0.323, *p* < 0.001) and its five subscales (*r* = 0.173–0.356) as well as PoM (*r* = 0.331, *p* < 0.001). Lastly, the subscales of resilience had association with PoM (*r* = 0.335–626), the total score of resilience had the largest association with PoM (*r* = 0.645, *p* < 0.0001).

**Figure 1:**
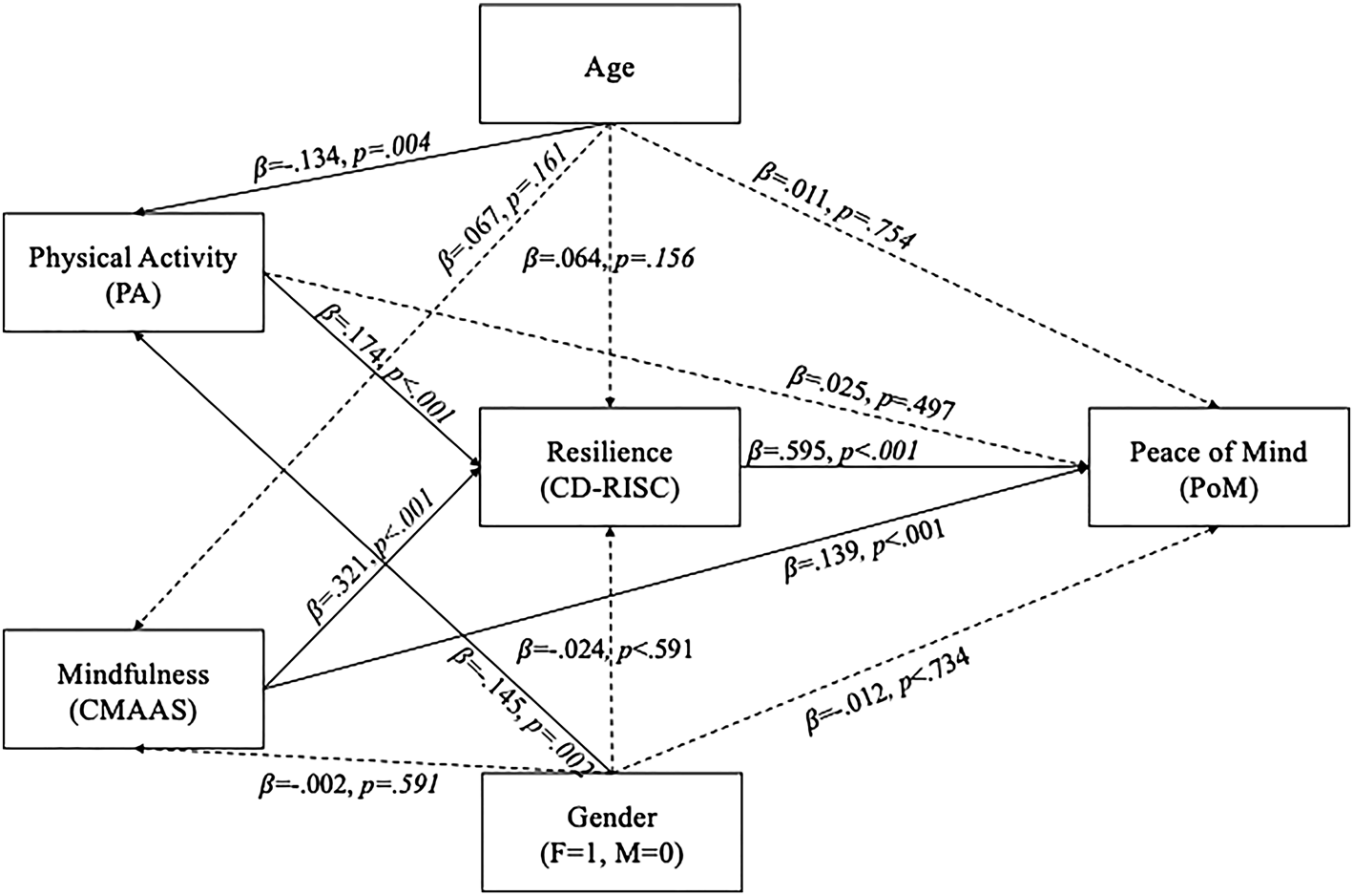
The path model’s standardized path coefficients regarding the age, gender, physical activity, mindfulness, resilience, and peace of mind variables. Standardized path coefficients among variables are presented. Solid lines were significant effects; dashed line was nonsignificant effect; F, female; M, male.

We used path analysis because scales for PA and PoM are unidimensional and we are interested in the overall resilience as the research concept, which is represented by the total score of the used resilience scale. We estimated the path model after controlling the effect of age and gender, in which physical activity and mindful self-awareness both predicted resilience, which in turn predicted PoM. Figure 1 presents the model and the standardized estimated effects. The solid lines were significant effects; dashed line was nonsignificant effect. The model fit the data well (χ^2^ = 0.130, df = 2; RMSEA = 0.000 (95% CI = 0.000–0.023), SRMR = 0.0039ss, CFI = 1.000, NNFI = 1.044) and supported our hypotheses.

Table 2 presents standardized estimates of direct and indirect effects in the path model. We found age and gender both have direct effects on physical activity ($\beta =$− 0.134, p < 0.004; $\beta \hspace{0.17em}$= − 0.145, *p* < 0.002), but these two variables had no direct effect on mindful self-awareness, resilience, and PoM. The physical activity and mindful self-awareness had indirect effects ($\beta \hspace{0.17em}=\hspace{0.17em}$0.104, p < 0.001; $\beta \hspace{0.17em}$= 0.191, *p* < 0.001) on PoM. Resilience mediated the link from physical activity to PoM and the link from mindful self-awareness to PoM. Mindful self-awareness also had a direct effect ($\beta \hspace{0.17em}=\hspace{0.17em}$0.139, *p* < 0.001) on PoM. However, physical activity had no direct effect ($\beta =$ 0.025, *p* > 0.05) on PoM.

**Table 2:** Standardized estimates of effects in the path model.

	Standardized effect ($\beta$)	95% CI
Age—> Physical activity	− .134**	[− .222, − .041]
Age—> Mindfulness	.067	[− .029, .160]
Age—> Resilience	.062	[− .027, .158]
Age—> Peace of mind	.054	[− .029, .142]
Gender—> Physical activity	− .145**	[− .234, − .051]
Gender—> Mindfulness	− .002	[− .098, .088]
Gender—> Resilience	− .050	[− .141, .046]
Gender—> Peace of mind	− .046	[− .142, .053]
Physical activity—> Resilience	.174***	[.082, .258]
Mindfulness—> Resilience	.321***	[.226, 409]
Physical activity—> Resilience—> Peace of mind	.104***	[.047, .157]
Physical activity—> Peace of mind	.025	[− .051, .099]
Mindfulness—> Resilience—> Peace of mind	.191***	[.134, .246]
Mindfulness—> Peace of mind	.139***	[.068, .215]
Resilience—> Peace of mind	.595***	[.515, .662]

*95% CI* = 95% confidence intervals. ***p < .001, **p < .01.

In the model, the standardized total effect ($\beta$) of mindful self-awareness on resilience was 0.321 (*p* = 0.001), physical activity on resilience was 0.174 (*p* = 0.001); mindful self-awareness on PoM was 0.330 (*p* = 0.001), physical activity on PoM was 0.129 (*p* = 0.003), and resilience on PoM was 0.595 (*p* = 0.001).

## Discussion

Our model revealed that increased age and being female negatively impact physical activity. However, these two variables did not affect mindful self-awareness, resilience, or peace of mind (PoM). The physical activity and mindful self-awareness can affect one's PoM through the mediating role of resilience. In the following, we discussed the three factors and their relationship.

First, mindful self-awareness affects PoM, such that how a person pays attention during everyday awareness may affect his or her PoM state through change resilience, including awareness of emotion, daily activity, automatic inattentiveness, or running on automatic pilot. The direct effect of mindful self-awareness on PoM was consistent with the mechanisms of mindfulness, which purport to intentionally cultivate nonjudgmental attention, lead to a connection with self-regulation and improve one’s mental health^10^. Mindful self-awareness, which emphasizes accepting the present moment in a peaceful and nonjudgmental manner and reducing immediate responsive behaviors in the face of adversity and stress, is essential for nurturing resilience^39,47–50^. This could explain the mediation effect of resilience between mindful self-awareness and PoM.

Second, our findings suggested that maintaining regular physical activity (over 150 min/week) did not affect PoM directly. This may be due to a reason that physical activity needs to firstly create changes at the biological, psychological and behavior activities to help promote well-being. According to Lubans et al.^51^, there are three potential mechanisms for physical activity with mental health outcomes: changes tbrain'sn'sn's structure and function, fulfillment of psychological needs for social connection, autonomy, self-acceptance, as well as changes in associated behaviors (such as improving sleep quality and self-regulation skills). Without having impact on these intervening mechanisms, individuals are unlikely to experience positive impact of physical activity. Similar to this notion, we found a complete mediation effect of resilience between physical activity and PoM, suggesting that physical activity affects one’s PoM state by improving resilience. In this manner, the frequency or intensity of physical activity might not directly improve one’s PoM or inner peace, it might work through improving the nervous system by increasing top-down cognitive control, and behavioral and emotional self-regulation for resilience^38,52^ , which helps individuals be more resistant to the emotional effects of acute stress^53^.

Third, the mediating role of resilience in this study suggests that resilience is potentially a crucial factor affecting the relationships among psychological and physical variables in health outcomes. Our results further showed all 5 subscales of resilience factor, including personal competence, trust, positive acceptance, control, and even spiritual influence had significant relation with PA, mindful self-awareness, and PoM. We found a mind–body integrated effect on enhancing a person’s resilience and contribution to one’s PoM. It might increase the ability to sufficiently control pathogenic factors, including pathogens and psychological stress or conflict^54^. In this manner, physical activity might affect PoM through resilience factors that improve one’s personal competence and sense of control. Mindful self-awareness affects PoM through improving resilience factors that increase one’s tolerance of negative affect acceptance of change^35^. Additionally, the body and mind interact together^56^. Thus, there is the potential to modulate neural systems related to self-specifying and narrative self-processing in a fashion that improves efficiency, integration, and flexibility to switch between systems of processing^55^.

To conclude, our study proposed a hybrid model, including the role of body-mind factors and resilience affecting PoM. We provide a valuable model for practical help in assessing and enhancing PoM. Although physical activity had no direct effects on PoM, it was a potential factor for enhancing PoM. This model suggests that regular physical activity and mindful self-awareness may positively affect resilience and PoM. We also emphasize the importance of body and mind exercise in improving resilience akeepione'se'se's PoM, specifically regular physical activities of more than 150 min/week and maintaining mindful self-awareness across daily life. By having more resources to manage stress, one might be better positioned to maintain and pursue resources to sustain the process of combatting stress and bouncing back from adversity to facilitate PoM.

## Limitations and future directions

Among this study's limitations was that the measurement of physical activity was subjectively recalled by participants and was only measured at the individual level, such that it reflected beliefs rather than an accurate indicator of moment-to-moment experiences. Future studies should use objective devices such as smartwatches and smartphone health apps.

Second, our model was constructed from a college sample; the age range only included individuals aged 18–30 years. Future studies may focus on increasing the generalizability of the sample by including older individuals or adapting different measures of mental health status.

Third, to our knowledge, few studies have focused on PoM outside of Asia. However, Fave et al.^56^ explored definitions of happiness in 12 countries, showing that the most frequently selected component of happiness was inner peace, as reported by over half of the participants from Italy, Brazil, and India. Our hybrid PoM model, which connects the body and mind variables keeping one's internal state of peacefulness and harmony, would be a practical concept of mental health that may extend to these cultural backgrounds.

Lastly, our study suggests a mediating role of psychological resilience in the mind–body model; however, future studies might consider the role of different types of resilience measurements. For example, physical resilience is one's ability to withstand or recover from functional decline following acute or chronic health stressors^57^. Thus, a future investigation might explore the role of mindful self-awareness and physical activity on physical resilience and potentially PoM. This study is based on a cross-sectional design; thus, how physical activity and mindful self-awareness work to enhance one’s resilience (including physical or psychological resilience) to improve PoM still needs to be investigated in a future follow-up study.

## Data Availability

The datasets analyzed during the current study are available in the GitHub repository (https://github.com/Kelvinycliao/super-octo-waffle.git).
